# RUNX1 and REXO2 are associated with the heterogeneity and prognosis of IDH wild type lower grade glioma

**DOI:** 10.1038/s41598-021-91382-1

**Published:** 2021-06-04

**Authors:** Haiwei Wang, Xinrui Wang, Liangpu Xu, Ji Zhang, Hua Cao

**Affiliations:** 1 Medical Research Center, Fujian Maternity and Child Health Hospital, Affiliated Hospital of Fujian Medical University, Fuzhou, Fujian China; 2 Key Laboratory of Technical Evaluation of Fertility Regulation for Non-Human Primate, National Health and Family Planning Commission, Fuzhou, Fujian China; 3 State Key Laboratory for Medical Genomics, Shanghai Institute of Hematology, Rui-Jin Hospital Affiliated to School of Medicine, Shanghai Jiao Tong University, Shanghai, China

**Keywords:** CNS cancer, Tumour heterogeneity

## Abstract

Based on isocitrate dehydrogenase (IDH) alterations, lower grade glioma (LGG) is divided into IDH mutant and wild type subgroups. However, the further classification of IDH wild type LGG was unclear. Here, IDH wild type LGG patients in The Cancer Genome Atlas and Chinese Glioma Genome Atlas were divided into two sub-clusters using non-negative matrix factorization. IDH wild type LGG patients in sub-cluster2 had prolonged overall survival and low frequency of CDKN2A alterations and low immune infiltrations. Differentially expressed genes in sub-cluster1 were positively correlated with RUNX1 transcription factor. Moreover, IDH wild type LGG patients with higher stromal score or immune score were positively correlated with RUNX1 transcription factor. RUNX1 and its target gene REXO2 were up-regulated in sub-cluster1 and associated with the worse prognosis of IDH wild type LGG. RUNX1 and REXO2 were associated with the higher immune infiltrations. Furthermore, RUNX1 and REXO2 were correlated with the worse prognosis of LGG or glioma. IDH wild type LGG in sub-cluster2 was hyper-methylated. REXO2 hyper-methylation was associated with the favorable prognosis of LGG or glioma. At last, we showed that, age, tumor grade and REXO2 expression were independent prognostic factors in IDH wild type LGG.

## Introduction

Lower grade glioma (LGG) is grade II-III glioma, contrast with grade IV glioma (glioblastoma, GBM)^1^. Genome wide analysis reveals that more than 80% of LGG^2^ and secondary GBM^3,4^ are characterized with isocitrate dehydrogenase 1 (IDH1) mutations. IDH2 mutations are less common, but also present in the glioma. Amino acid mutation at arginine 132 (R132) confers a new metabolic activity of IDH1 which catalyzes α-ketoglutarate to 2-hydroxyglutarate (2HG)^5^. IDH mutation induces massive DNA hyper-methylation^6^ and chromatin alterations^7,8^ to promote glioma formation^9,10^. Clinically, based on the characterizations of IDH, LGG patients are classified into IDH wild type LGG and IDH mutant LGG subgroups. IDH mutant LGG is associated with prolonged overall survival^11–13^ and increased sensitivity to temozolomide therapy^14^ and radiation therapy^15–17^.

Furthermore, LGG harboring IDH mutations is heterogeneous and could be divided into two molecular sub-types. One sub-type contains 1p/19q codeletion and TERT promoter mutation and the other sub-type contains TP53 mutation and inactivated α-thalassemia/mental retardation syndrome X-linked (ATRX) gene^18,19^. LGG with IDH mutation along with 1p/19q codeletion has ever better clinical outcomes^11,12^. IDH wild type LGG is also heterogeneous and has varied prognosis^20,21^. IDH wild type LGG patients with TERT promoter mutation, EGFR amplification or H3F3A mutation have ever worse clinical outcomes^22^. Moreover, CDKN2A deletion, TERT mutation and EGFR amplification are associated with the prognostic outcomes of IDH wild type GBM^23^.

Except genetic alterations, transcriptional clustering of “ConsensusClusterPlus”^24,25^ and “non-negative matrix factorization (NMF)”^26,27^ are robust methods to reveal the cancer molecular heterogeneity. Using “ConsensusClusterPlus” classification, IDH wild type LGG is divided into two sub-types with distinct clinical outcomes and immune alterations^28^. However, the NMF classification of LGG patients in each IDH wild type or IDH mutant core subgroup is unclear.

In this paper, based on the global transcriptional data deposited in The Cancer Genome Atlas (TCGA)^19^ and Chinese Glioma Genome Atlas (CGGA)^29,30^, we determined the inner heterogeneity of IDH wild type LGG patients. We found that IDH wild type LGG was a heterogeneous cohort, comprised two sub-clusters with different clinical outcomes, genetic alterations and immune alterations. RUNX1 was a critical factor determining the different transcriptional features of sub-clusters of IDH wild type LGG. RUNX1 was also associated with the immune alterations in IDH wild type LGG. Moreover, RUNX1 and its target gene REXO2 were associated with the poor prognosis of LGG, particularly of IDH wild type LGG patients. Our analysis provided novel prognostic makers to predict the clinical outcomes of LGG.

## Results

### Two molecular sub-clusters of IDH wild type LGG patients with different clinical outcomes, genetic alterations and immune alterations

**Figure 1:**
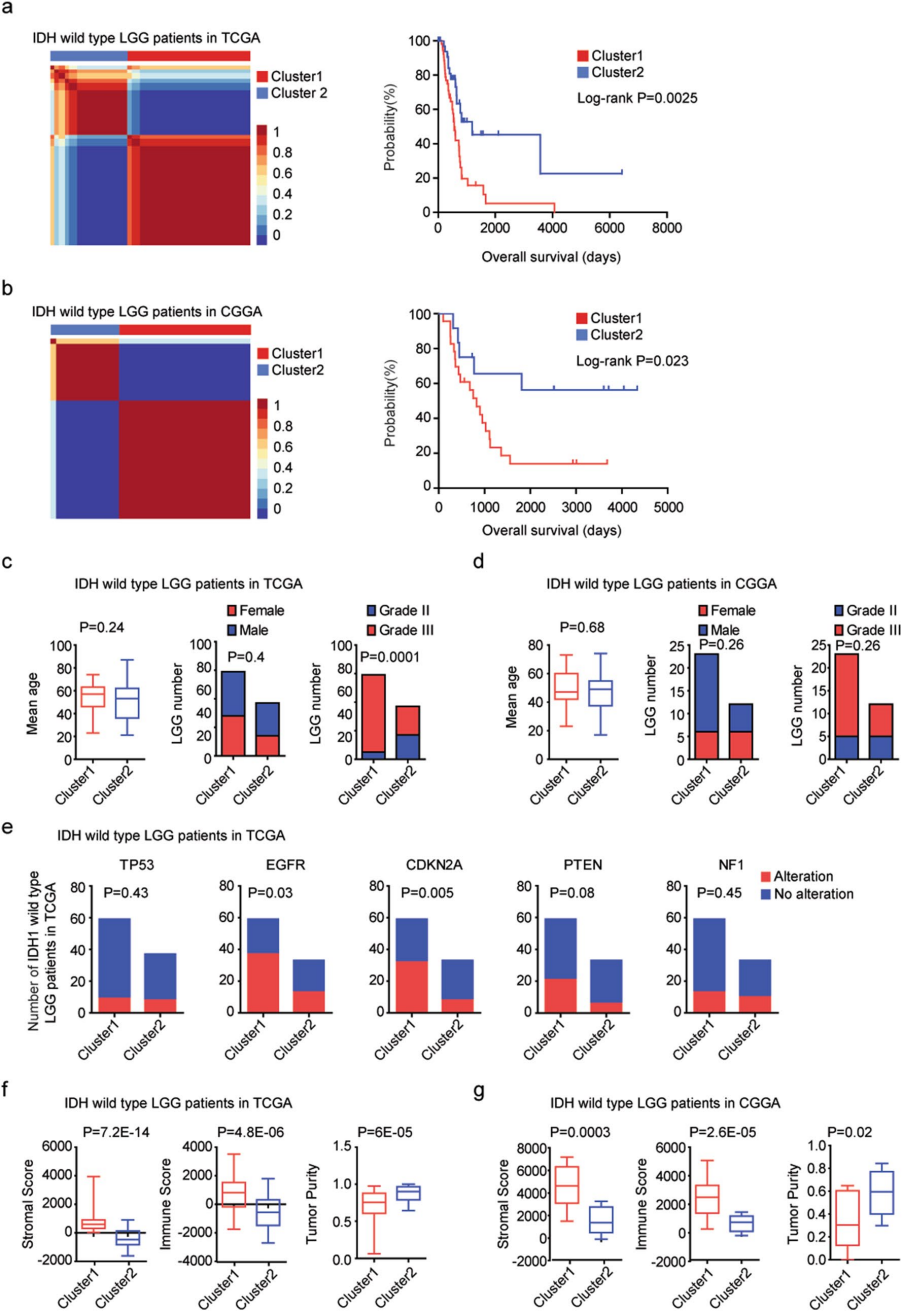
Two molecular sub-clusters of IDH wild type LGG patients with different clinical outcomes, genetic alterations and immune alterations. (**a**) Consensus-map showed that primary LGG patients without IDH mutations from TCGA dataset were divided into two sub-clusters using NMF method based on the all expressed genes. Kaplan–Meier survival plot showed the different overall survival of IDH wild type LGG patients in sub-cluster1 and sub-cluster2. *P* values were determined by log-rank test. (**b**) LGG patients without IDH mutations from CGGA dataset were divided into two sub-clusters using NMF method. The clinical outcomes of sub-cluster1 and sub-cluster2 were determined. (**c**) The difference of age, gender and tumor grade in the two sub-clusters of IDH wild type LGG patients from TCGA dataset. (**d**) The difference of age, gender and tumor grade in the two sub-clusters of IDH wild type LGG patients from CGGA dataset. (**e**) The number of IDH wild type LGG patients with or without TP53, EGFR, CDKN2A, PTEN, NF1 alterations in each sub-cluster. *P* values were calculated by Chi-square test. (**f**) Box plots showed the stromal score, immune score and tumor purity in sub-cluster1 and sub-cluster2 IDH wild type LGG patients in TCGA dataset. (**g**) Box plots showed the stromal score, immune score and tumor purity in sub-cluster1 and sub-cluster2 IDH wild type LGG patients in CGGA dataset.

To further address the inner sub-clusters of IDH wild type LGG, 96 LGG patients without IDH1 or IDH2 mutation from TCGA dataset were selected for further studies. Based on the global transcriptional profiling, those IDH wild type LGG patients were divided into two sub-clusters using “NMF” package in R software. 59 patients were classified into sub-cluster1 and 37 patients were classified into sub-cluster2 (Fig. 1a). The overall survival between sub-cluster1 and sub-cluster2 was significantly different. IDH wild type LGG patients in sub-cluster2 had increased overall survival than IDH wild type LGG patients in sub-cluster1 (*P* = 0.0025) (Fig. 1a). The two sub-clusters of NMF classification of IDH wild type LGG was tested using an independent CGGA dataset. 35 IDH wild type LGG patients were divided into two sub-clusters. 23 LGG patients were in sub-cluster1, and 12 LGG patients were in sub-cluster2, respectively (Fig. 1b). Compared with IDH wild type LGG patients in sub-cluster2, IDH wild type LGG patients in sub-cluster1 had more unfavorable clinical overall survival (*P* = 0.023) (Fig. 1b).

Moreover, in TCGA dataset, most patients in sub-cluster1 were grade III IDH wild type LGG (*P* = 0.0001) (Fig. 1c). However, there was no age or gender difference in the two sub-clusters of IDH wild type LGG patients in TCGA dataset (Fig. 1c). Also, there was no significant difference of age, gender and tumor grade in the two sub-clusters of IDH wild type LGG patients in CGGA dataset (Fig. 1d). Furthermore, compared with IDH wild type LGG patients in sub-cluster2, IDH wild type LGG patients in sub-cluster1 had higher frequency of EGFR and CDKN2A alterations in TCGA dataset (Fig. 1e). However, the TP53, PTEN and NF1 mutant frequency in sub-cluster1 and sub-cluster2 IDH wild type LGG patients was not significantly different (Fig. 1e).

Immune alterations were associated with the clinical outcomes and immunotherapy of IDH wild-type LGG^28^. Next, we tested the difference of immune alterations between the two sub-clusters. The stromal score and immune score of the IDH wild-type LGG in TCGA and CGGA datasets were determined using “ESTIMATE” package in R. We found that, compared with IDH wild type LGG patients in sub-cluster2, IDH wild type LGG patients in sub-cluster1 had higher stromal score (*P* = 7E−11) and immune score (*P* = 2.6E−15) in TCGA dataset (Fig. 1f). Similarly, in CGGA dataset, the stromal score and immune score were higher in sub-cluster1 IDH wild type LGG patients (*P* = 0.0003 and *P* = 2.6E−05, respectively) (Fig. 1g). The IDH wild type LGG patients in sub-cluster1 were with lower tumor purity in TCGA (*P* = 5.4E−10) (Fig. 1f) and CGGA (Fig. 1g) (*P* = 0.02) datasets. Those results suggested that IDH wild type LGG was a heterogeneous cohort, and could be further divided into two sub-clusters with different clinical outcomes, genetic alterations and immune alterations.

### Transcriptional characteristics of the different sub-clusters of IDH wild type LGG patients

**Figure 2:**
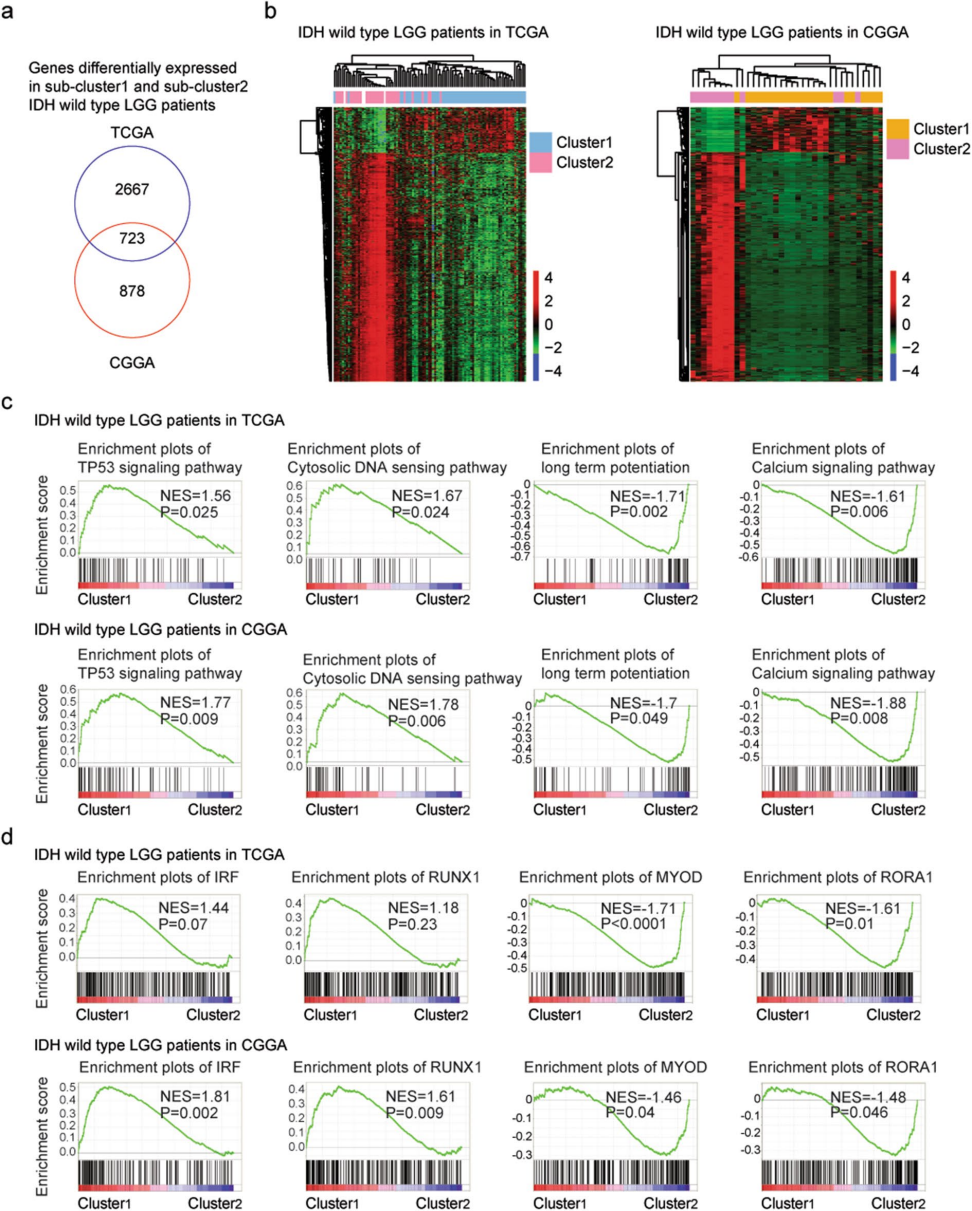
Transcriptional characteristics of the different sub-clusters of IDH wild type LGG patients. (**a**) Overlapping the differentially expressed genes between sub-cluster1 and sub-cluster2 IDH wild type LGG patients in TCGA and CGGA datasets. (**b**) Un-supervised clustering heatmaps demonstrated the common differentially expressed genes between sub-cluster1 and sub-cluster2 IDH wild type LGG patients in TCGA and CGGA datasets. Red represented the up-regulated genes and blue represented the down-regulated genes. (**c**) Enrichment plots showed the enriched signaling pathways in different sub-clusters of IDH wild type LGG patients in TCGA and CGGA datasets. (**d**) Enriched transcription factors in different sub-clusters of IDH wild type LGG patients in TCGA and CGGA datasets. (**e**) Enrichment plots of RUNX1 transcription factor in different stromal score and immune score IDH wild type LGG patients in TCGA and CGGA datasets.

Based on the criterion of absolute fold change > 2 and *P* values < 0.001, the differentially expressed genes between sub-cluster1 and sub-cluster2 of IDH wild type LGG patients were identified. These resulted 3390 differentially expressed genes in TCGA dataset and 1601 differentially expressed genes in CGGA dataset, respectively. 723 genes were commonly changed between sub-cluster1 and sub-cluster2 IDH wild type LGG patients in TCGA and CGGA datasets (Fig. 2a). Un-supervised clustering heatmaps showed that those genes divided the IDH wild type LGG patients into two distinct subgroups and most genes were up-regulated in sub-cluster2 IDH wild type LGG patients (Fig. 2b).

Using gene set enrichment analysis (GSEA), we further determined the Kyoto Encyclopedia of Genes and Genomes (KEGG) signaling pathways associated with the transcriptional signatures in sub-cluster1 and sub-cluster2 IDH wild type LGG patients in TCGA and CGGA datasets. Based on the criterion of *P* values < 0.05, we found that IDH wild type LGG patients in sub-cluster1 were positively correlated with TP53 signaling pathway and cytosolic DNA sensing signaling pathway, while, negatively correlated with long term potentiation and calcium signaling pathway in TCGA and CGGA datasets (Fig. 2c).

Also, transcription factors associated with the transcriptional signatures of sub-cluster1 and sub-cluster2 IDH wild type LGG patients in TCGA and CGGA datasets were identified. IDH wild type LGG patients in sub-cluster1 were positively correlated with IRF and AML1 (RUNX1) transcription factors, while, negatively correlated with MYOD and RORA1 transcription factors in CGGA datasets (Fig. 2d). However, the enrichment of IRF and RUNX1 transcription factors in sub-cluster1 IDH wild type LGG patients was not significant in TCGA dataset (*P* = 0.07 and *P* = 0.23, respectively) (Fig. 2d).

### Transcriptional characteristics of of IDH wild type LGG patients with different immune infiltrations

**Figure 3:**
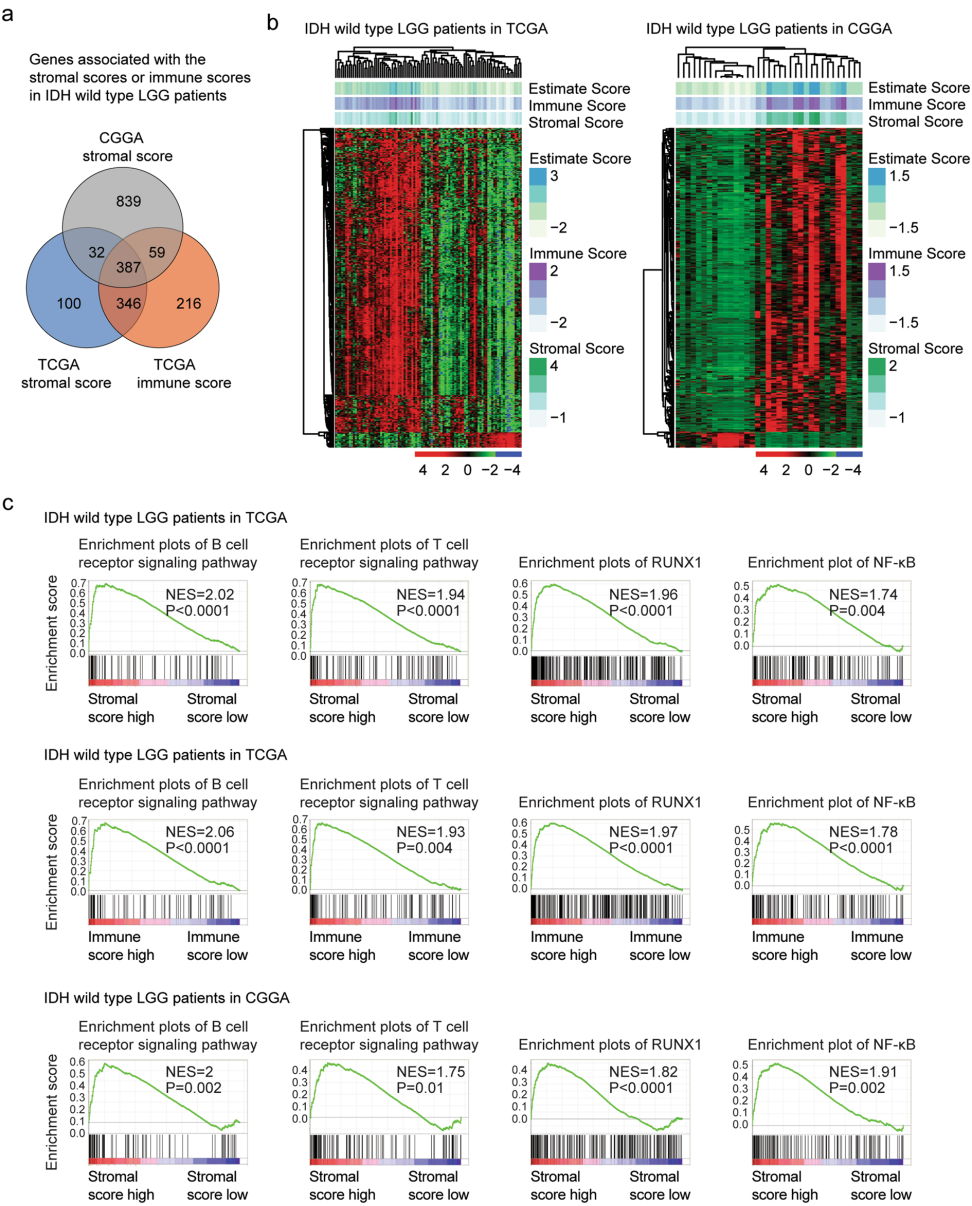
Transcriptional characteristics of of IDH wild type LGG patients with different immune infiltrations. (**a**) Overlapping the differentially expressed genes in IDH wild type LGG patients with different immune infiltrations in TCGA and CGGA datasets. (**b**) Un-supervised clustering heatmaps demonstrated the common differentially expressed genes in IDH wild type LGG patients with different immune infiltrations in TCGA and CGGA datasets. (**c**) Enrichment plots of B cell receptor signaling pathway, T cell receptor signaling pathway and RUNX1, NF-κB transcription factors in different stromal score and immune score IDH wild type LGG patients in TCGA and CGGA datasets.

Genes associated with the immune alterations in IDH wild type LGG patients were also determined. The normalized stromal score or immune score > 0 were defined as high stromal score or immune score. Based on the criterion of absolute fold change > 2 and *P* values < 0.001, 865 genes were associated with high stromal score, while, 1008 genes were associated with high immune score in TCGA dataset (Fig. 3a). In CGGA dataset, IDH wild type LGG patients with high stromal score were also with high immune score. We identified 1317 genes were associated with the immune alterations in CGGA dataset (Fig. 3a). 387 genes were differentially expressed in IDH wild type LGG patients in with different immune infiltrations in TCGA and CGGA datasets (Fig. 3a). Un-supervised clustering heatmaps showed that those genes divided the IDH wild type LGG patients into two distinct subgroups and most genes were up-regulated in IDH wild type LGG patients with higher immune infiltrations (Fig. 3b).

Furthermore, the KEGG signaling pathways and transcription factors associated with the immune alterations of IDH wild type LGG patients were determined. B cell receptor signaling pathway and T cell receptor signaling pathway were significantly positively enriched in IDH wild type LGG patients with high stromal score or immune score in TCGA and CGGA datasets (Fig. 3c). Moreover, RUNX1 and NF-κB transcription factors were significantly associated with the stromal score and immune score in IDH wild type LGG patients. IDH wild type LGG patients with higher stromal score or immune score were positively correlated with RUNX1 and NF-κB transcription factors in TCGA and CGGA datasets (Fig. 3c).

### Transcription factor RUNX1 is up-regulated in sub-cluster1 and associated with the worse clinical outcomes of IDH wild type or IDH mutant LGG patients

**Figure 4:**
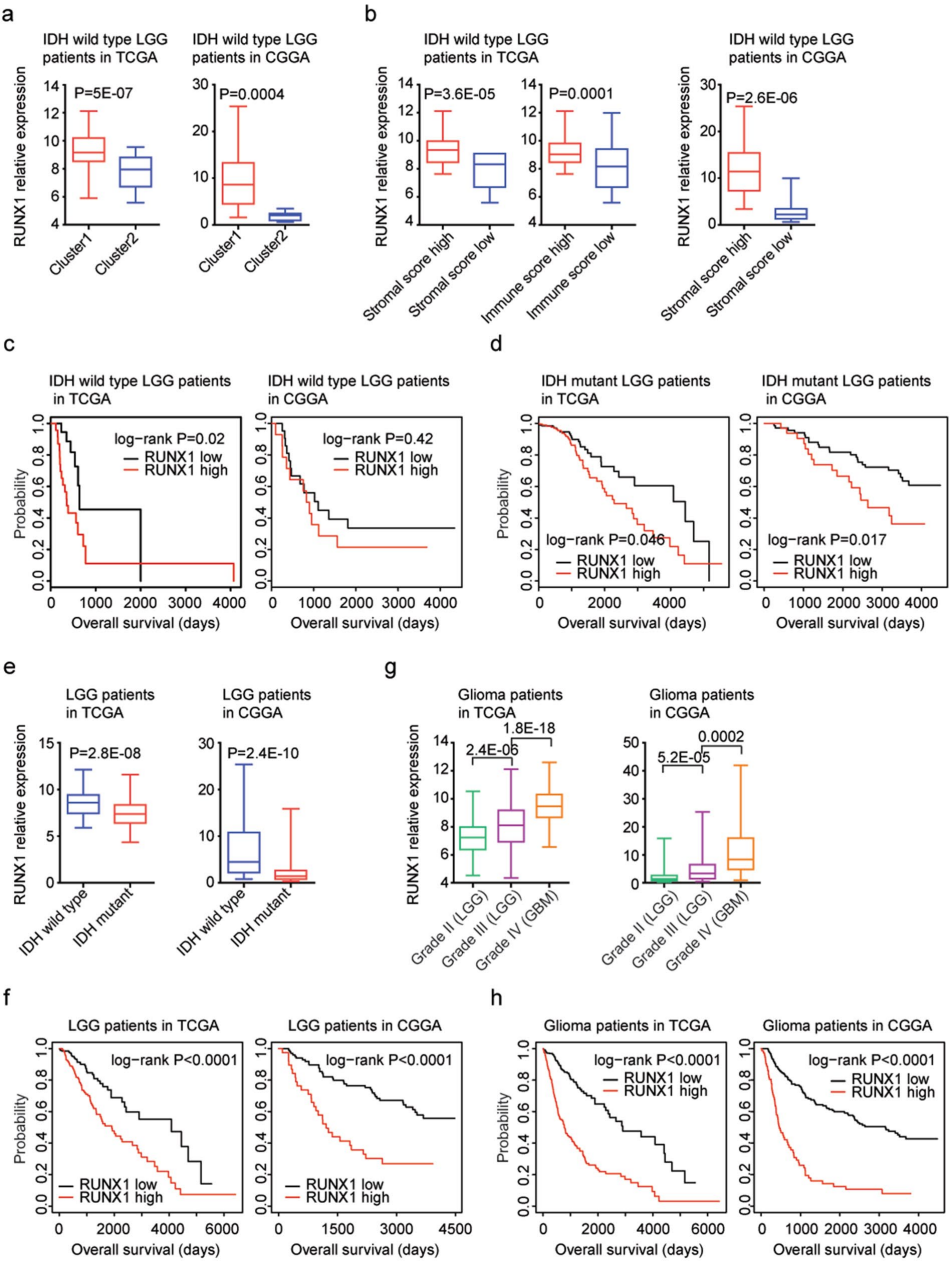
Transcription factor RUNX1 is up-regulated in sub-cluster1 and associated with the worse clinical outcomes of IDH wild type or IDH mutant LGG or glioma patients. (**a**) Box plots showed the expression levels of RUNX1 in sub-cluster1 and sub-cluster2 IDH wild type LGG patients in TCGA and CGGA datasets. *P* values were determined by two tails paired Student’s t test. (**b**) Box plots showed the RUNX1 expression levels in TCGA and CGGA IDH wild type LGG patients with higher stromal or immune score (red) or with lower stromal or immune score (blue). (**c**) The Kaplan–Meier Plotters showed the associations between RUNX1 expression and overall survival in IDH wild type LGG patients in TCGA and CGGA datasets. *P* values showed the different overall survival between RUNX1 highly expressed IDH wild type LGG patients (red) and RUNX1 lowly expressed IDH wild type LGG patients (black). (**d**) The Kaplan–Meier Plotters showed the associations between RUNX1 expression and overall survival in IDH mutant LGG patients in TCGA and CGGA datasets. (**e**) The expression levels of RUNX1 in LGG patients with (red) or without (blue) IDH mutations in TCGA and CGGA datasets. (**f**) The Kaplan–Meier Plotters showed the correlations of RUNX1 expression levels and LGG overall survival in TCGA and CGGA datasets. (**g**) Box plots demonstrated the expression levels of RUNX1 in grade II (LGG), grade III (LGG) or grade IV (GBM) glioma patients in TCGA and CGGA datasets. (**h**) The Kaplan–Meier Plotters showed the correlations of RUNX1 expression levels and glioma overall survival in TCGA and CGGA datasets.

RUNX1 was reported to be a prognostic factor in high grade glioma (GBM)^31^. However, the prognosis of RUNX1 in lower grade glioma (LGG), particularly in IDH wild type LGG was unknown. So, the expression levels and prognostic significance of RUNX1 in IDH wild type LGG were further tested. First, we found that, compared with sub-cluster2 IDH wild type LGG patients, RUNX1 was up-regulated in sub-cluster1 IDH wild type LGG patients in TCGA (*P* = 5E−07) and CGGA (*P* = 0.0004) datasets (Fig. 4a). Moreover, RUNX1 was up-regulated in IDH wild type LGG patients with higher stromal score, compared with IDH wild type LGG patients with lower stromal score in TCGA (*P* = 3.6E−05) and CGGA (*P* = 2.6E−06) datasets (Fig. 4b). Also, compared with IDH wild type LGG patients with lower immune score, the expression levels of RUNX1 were higher in IDH wild type LGG patients with higher immune score in TCGA (*P* = 0.0001) datasets (Fig. 4b).

Based on the mean expression value of RUNX1, IDH wild type LGG patients were divided into higher RUNX1 expression or lower RUNX1 expression subgroups. Corresponding to the higher expression levels of RUNX1 in sub-cluster1 IDH wild type LGG patients, higher RUNX1 expression was associated with the worse prognosis of IDH wild type LGG patients in TCGA dataset (*P* = 0.02) (Fig. 4c). However, in CGGA dataset, there was no significantly different clinical overall survival between RUNX1 highly or lowly expressed IDH wild type LGG patients (*P* = 0.42) (Fig. 4c). Interestingly, RUNX1 expression was also a prognostic factor in IDH mutant LGG patients. Compared with RUNX1 highly expressed IDH mutant LGG patients, RUNX1 lowly expressed IDH mutant LGG patients had more favorable clinical overall survival in TCGA (*P* = 0.046) and CGGA (*P* = 0.017) datasets (Fig. 4d).

### RUNX1 expression is associated with IDH mutation and the worse clinical outcomes of LGG or glioma patients

Moreover, we showed that RUNX1 expression was associated with IDH mutation. Compared with IDH wild type LGG patients, RUXN1 was down-regulated in IDH mutant LGG patients in TCGA (*P* = 2.8E−08) and CGGA (*P* = 2.4E−10) datasets (Fig. 4e). Moreover, RUNX1 expression levels were correlated with the LGG overall survival in both TCGA (*P* < 0.0001) and CGGA (*P* < 0.0001) datasets (Fig. 4f). Overall survival was increased in RUNX1 lowly expressed LGG patients, compared with RUNX1 highly expressed LGG patients (Fig. 4f).

Compared with grade II LGG patients, RUNX1 was highly expressed in grade III LGG patients in TCGA (*P* = 2.4E−06) and CGGA (*P* = 5.2E−05) datasets (Fig. 4g). Furthermore, RUNX1 was further over-expressed in grade IV glioma (GBM) patients in TCGA (*P* = 1.8E−18) and CGGA (*P* = 0.002) datasets (Fig. 4g), suggested the increased expressions of RUNX1 with the increased malignance of glioma. We further tested the prognostic significance of RUNX1 in glioma patients. We found that RUNX1 highly expressed glioma had lower overall survival than RUNX1 lowly expressed glioma patients in TCGA (*P* < 0.0001) and CGGA (*P* < 0.0001) datasets (Fig. 4h).

### RUNX1 target gene REXO2 is up-regulated in sub-cluster1 and associated with the worse clinical outcomes of IDH wild type or IDH mutant LGG patients

**Figure 5:**
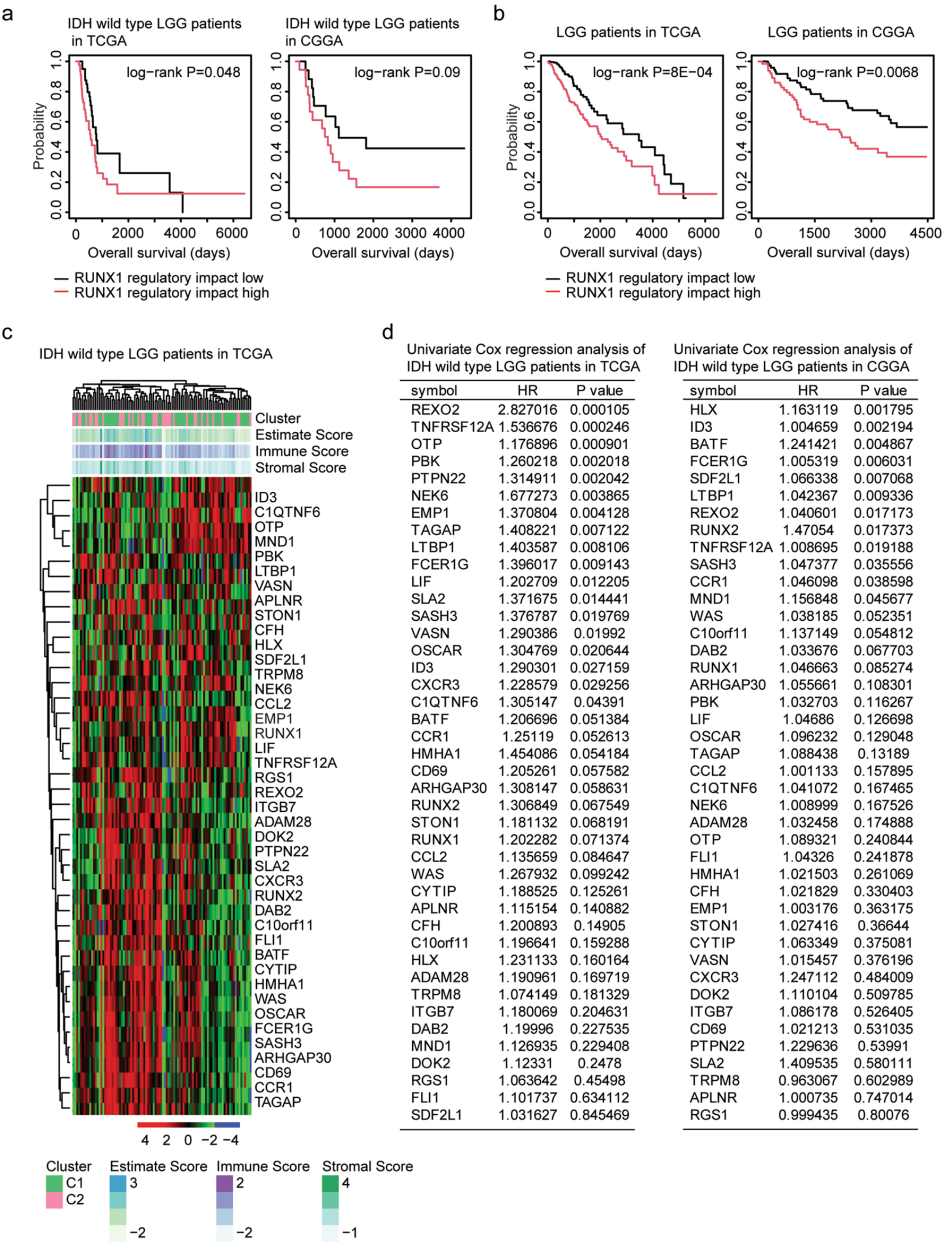
Prognostic significance of RUNX1 target genes. (**a**) The Kaplan–Meier Plotters showed the correlations of the regulatory impact of RUNX1 and overall survival of IDH wild type LGG in TCGA and CGGA datasets. (**b**) The Kaplan–Meier Plotters showed the correlations of the regulatory impact of RUNX1 and overall survival of LGG in TCGA and CGGA datasets. (**c**) Un-supervised clustering heatmaps demonstrated the expression of RUNX1 target genes in IDH wild type LGG patients in TCGA dataset. (**d**) Univariate cox regression analysis showed the prognostic significance of RUNX1 target genes in IDH wild type LGG patients in TCGA and CGGA datasets. HR represented hazard ratio.

As a transcription factor, RUNX1 regulates the expression levels of multiple target genes^32,33^. The regulatory impact of RUNX1 was determined using single sample gene set enrichment analysis (ssGSEA) in R “GSVA” package. We found that IDH wild type LGG patients with lower regulatory impact of RUNX1 had increased overall survival than IDH wild type LGG patients with higher regulatory impact of RUNX1 in TCGA dataset (*P* = 0.048) (Fig. 5a). However, the regulatory impact of RUNX1 was not associated with the overall survival of IDH wild type LGG patients in CGGA dataset (*P* = 0.09) (Fig. 5a). Furthermore, the regulatory impact of RUNX1 was correlated with the LGG overall survival in both TCGA (*P* = 8E−04) and CGGA (*P* = 0.0068) datasets (Fig. 5b).

Using the GSEA gene sets, we identified 42 RUNX1 target genes. Un-supervised clustering heatmaps demonstrated that most of the RUNX1 target genes were up-regulated in sub-cluster1 IDH wild type LGG patients in TCGA dataset (Fig. 5c). Furthermore, the prognostic effects of RUNX1 target genes were determined using univariate cox regression analysis. Six genes REXO2, FCER1G, ID3, LTBP1, SASH3 and TNFRSF12A were associated with the prognosis of IDH wild type LGG patients in TCGA and CGGA datasets (Fig. 5d). REXO2 was most correlated with the overall survival of IDH wild type LGG patients in TCGA dataset (*P* = 0.0001) and was the seventh gene most correlated with the overall survival of IDH wild type LGG patients in CGGA dataset (*P* = 0.017) (Fig. 5d). So, we focused on REXO2 for our next studies.

**Figure 6:**
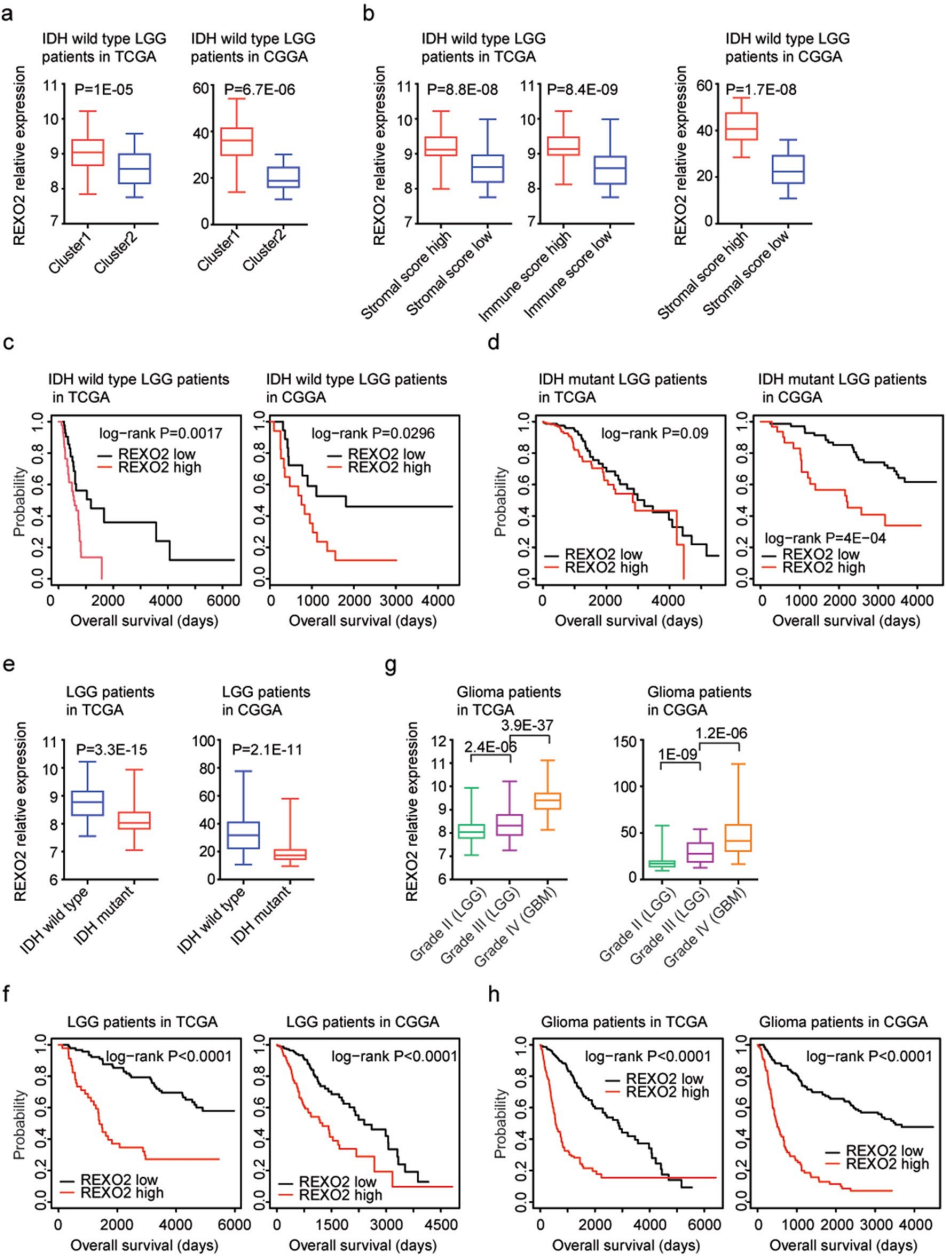
REXO2 is up-regulated in sub-cluster1 and associated with the worse clinical outcomes of IDH wild type or IDH mutant LGG or glioma patients. (**a**) Box plots showed the expression levels of REXO2 in sub-cluster1 and sub-cluster2 IDH wild type LGG patients in TCGA and CGGA datasets. (**b**) Box plots showed the REXO2 expression levels in TCGA and CGGA IDH wild type LGG patients with higher stromal or immune score (red) or with lower stromal or immune score (blue). (**c**) The Kaplan–Meier Plotters showed the overall survival of REXO2 highly expressed IDH wild type LGG patients (red) and REXO2 lowly expressed IDH wild type LGG patients (black) in TCGA and CGGA datasets. (**d**) The Kaplan–Meier Plotters showed the associations between REXO2 expression and overall survival in IDH mutant LGG patients in TCGA and CGGA datasets. (**e**) The expression levels of REXO2 in LGG patients with (red) or without (blue) IDH mutations in TCGA and CGGA datasets. (**f**) The Kaplan–Meier Plotters showed the correlations of REXO2 expression levels and LGG overall survival in TCGA and CGGA datasets. (**g**) Box plots demonstrated the expression levels of REXO2 in grade II (LGG), grade III (LGG) or grade IV (GBM) glioma patients in TCGA and CGGA datasets. (**h**) The Kaplan–Meier Plotters showed the correlations of REXO2 expression levels and glioma overall survival in TCGA and CGGA datasets.

REXO2 is a RNA binding protein, and the prognostic effects of REXO2 in LGG were unknown. Here, we showed that, like RUNX1, REXO2 was up-regulated in sub-cluster1 IDH wild type LGG patients in TCGA (*P* = 1E−05) and CGGA (*P* = 6.7E−06) datasets (Fig. 6a). Also, REXO2 was up-regulated in IDH wild type LGG patients with higher stromal score or immune score, compared with IDH wild type LGG patients with lower stromal score or immune score in TCGA and CGGA datasets (Fig. 6b). Moreover, higher REXO2 expression was associated with the worse prognosis of IDH wild type LGG patients in TCGA (*P* = 0.0017) and CGGA (*P* = 0.0296) datasets (Fig. 6c). However, unlike RUNX1, REXO2 had no prognostic effects in IDH mutant LGG patients in TCGA dataset (*P* = 0.09) (Fig. 6d). On the contrary, in CGGA dataset, higher REXO2 expression was associated with the worse prognosis of IDH mutant LGG patients (*P* = 4E−04) (Fig. 6d).

### REXO2 expression is associated with IDH mutation and the worse clinical outcomes of LGG or glioma patients

REXO2 expression was also associated with IDH mutation. Compared with IDH wild type LGG patients, REXO2 was down-regulated in IDH mutant LGG patients in TCGA (*P* = 3.3E−05) and CGGA (*P* = 2.1E−11) datasets (Fig. 6e). Moreover, REXO2 expression levels were correlated with the overall survival of LGG in both TCGA (*P* < 0.0001) and CGGA (*P* < 0.0001) datasets (Fig. 6f). Overall survival was increased in REXO2 lowly expressed LGG patients, compared with REXO2 highly expressed LGG patients (Fig. 6f).

Compared with grade II LGG patients, REXO2 was highly expressed in grade III LGG patients in TCGA (*P* = 2.4E−06) and CGGA (*P* = 1E−09) datasets (Fig. 6g). Furthermore, REXO2 was further over-expressed in grade IV glioma (GBM) patients in TCGA (*P* = 3.9E−37) and CGGA (*P* = 1.2E−06) datasets (Fig. 6g). We also tested the prognostic significance of REXO2 in glioma patients. We found that REXO2 highly expressed glioma had lower overall survival than REXO2 lowly expressed glioma patients in TCGA (*P* < 0.0001) and CGGA (*P* < 0.0001) datasets (Fig. 6h).

### RUNX1 and REXO2 hyper-methylation are associated with the favorable clinical outcomes of IDH wild type LGG patients

**Figure 7:**
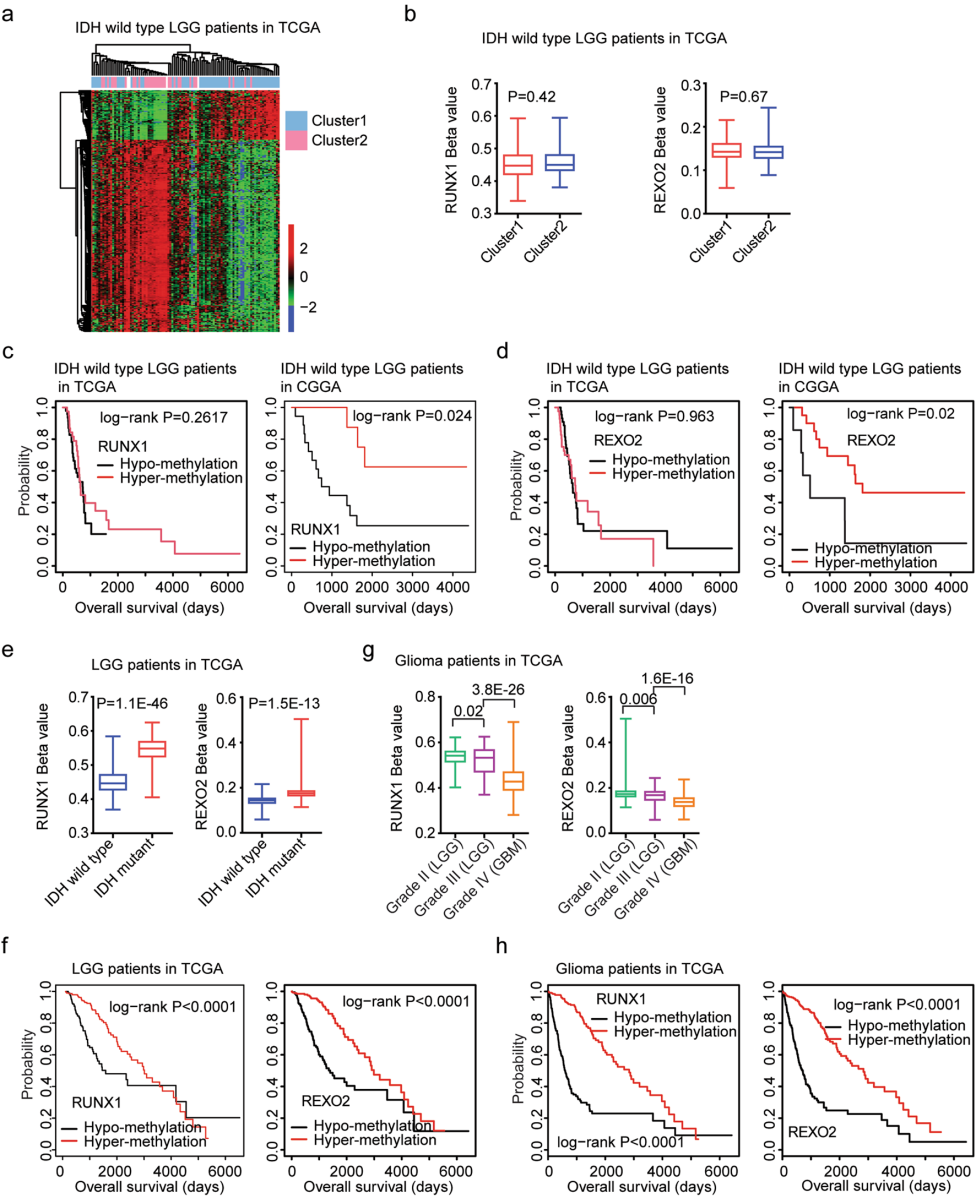
RUNX1 and REXO2 hyper-methylation are associated with the favorable clinical outcomes of IDH wild type LGG or LGG or glioma patients. (**a**) Un-supervised clustering heatmaps demonstrated the differentially methylated genes between sub-cluster1 and sub-cluster2 IDH wild type LGG patients in TCGA dataset. Red represented the hyper-methylated genes and blue represented the hypo-methylated genes. (**b**) Box plots showed the methylation levels of RUNX1 and REXO2 in sub-cluster1 and sub-cluster2 IDH wild type LGG patients in TCGA dataset. (**c**) The Kaplan–Meier Plotters demonstrated the different clinical outcomes of IDH wild type LGG patients with RUNX1 hypo-methylation or with RUNX1 hyper-methylation in TCGA and CGGA datasets. (**d**) The Kaplan–Meier Plotters demonstrated the different clinical outcomes of IDH wild type LGG patients with REXO2 hypo-methylation or with REXO2 hyper-methylation in TCGA and CGGA datasets. (**e**) Box plots showed the methylation levels of RUNX1 and REXO2 in LGG patients with (red) or without (blue) IDH mutations in TCGA dataset. (**f**) The Kaplan–Meier Plotters showed the correlations of RUNX1, REXO2 methylation levels and LGG overall survival in TCGA dataset. (**g**) Box plots demonstrated the methylation levels of RUNX1 and REXO2 in grade II (LGG), grade III (LGG) or grade IV (GBM) glioma patients in TCGA dataset. (**h**) The Kaplan–Meier Plotters showed the correlations of RUNX1, REXO2 methylation levels and glioma overall survival in TCGA dataset.

We had shown the different genetic alterations, clinical outcomes and transcriptional features between sub-cluster1 and sub-cluster2 IDH wild type LGG patients. Moreover, the DNA methylation profiling in IDH wild type LGG patients in sub-cluster1 or sub-cluster2 was also significantly different. Based on the criterion of the changes of beta values > 0.1 and *P* values < 0.001, 214 genes were hyper-methylated in sub-cluster2 IDH wild type LGG patients, while, only 55 genes were hyper-methylated in sub-cluster1 IDH wild type LGG patients (Fig. 7a). However, the methylation levels of RUNX1 and REXO2 were not significantly different in sub-cluster1 and sub-cluster2 IDH wild type LGG patients (*P* = 0.42 and *P* = 0.67 respectively) (Fig. 7b).

Next, we determined the prognostic effects of RUNX1 and REXO2 methylation levels. Based on the mean methylation values of RUNX1, IDH wild type LGG patients were divided into hypo-methylation or hyper-methylation subgroups. Kaplan–Meier Plotters demonstrated the clinical outcomes of IDH wild type LGG with RUNX1 hypo-methylation or with RUNX1 hyper-methylation. RUNX1 hyper-methylated IDH wild type LGG patients had lower overall survival than RUNX1 hypo-methylated IDH wild type LGG patient in CGGA dataset (*P* = 0.024), but not in TCGA dataset (*P* = 0.26) (Fig. 7c). And IDH wild type LGG patients with REXO2 hypo-methylation had better clinical outcomes than LGG patients with REXO2 hyper-methylation in CGGA dataset (*P*-0.02) (Fig. 7d). However, in TCGA dataset, the overall survival of IDH wild type LGG patients with REXO2 hypo-methylation or with REXO2 hyper-methylation was not significantly different (*P* = 0.963) (Fig. 7d).

### RUNX1 and REXO2 methylation are associated with IDH mutation and the better clinical outcomes of LGG or glioma patients

The 2HG accumulation in IDH mutant LGG could increase extensive epigenetic DNA methylation^6^ and maintain the CpG island methylator phenotype (CIMP)^34^. We showed that RUNX1 and REXO2 methylation levels were associated with IDH mutation. Compared with IDH wild type LGG patients, RUNX1 and REXO2 were hyper-methylated in IDH mutant LGG patients in TCGA dataset (*P* = 1.1E−46 and *P* = 1.5E−13, respectively) (Fig. 7e). Moreover, LGG patients with RUNX1 or REXO2 hypo-methylation had better clinical outcomes than LGG patients with RUNX1 or REXO2 hyper-methylation in TCGA dataset (*P* < 0.0001) (Fig. 7f).

Furthermore, compared with grade II LGG patients, RUNX1 and REXO2 were hypo-methylated in grade III LGG patients in TCGA dataset (Fig. 7g). RUNX1 and REXO2 were further hypo-methylated in grade IV glioma (GBM) patients in TCGA dataset (Fig. 7g). Glioma patients with RUNX1 or REXO2 hypo-methylation had worse clinical outcomes than LGG patients with RUNX1 or REXO2 hyper-methylation in TCGA dataset (Fig. 7h).

### Age, tumor grade and REXO2 expression are independent prognostic factors in IDH wild type LGG patients

**Figure 8:**
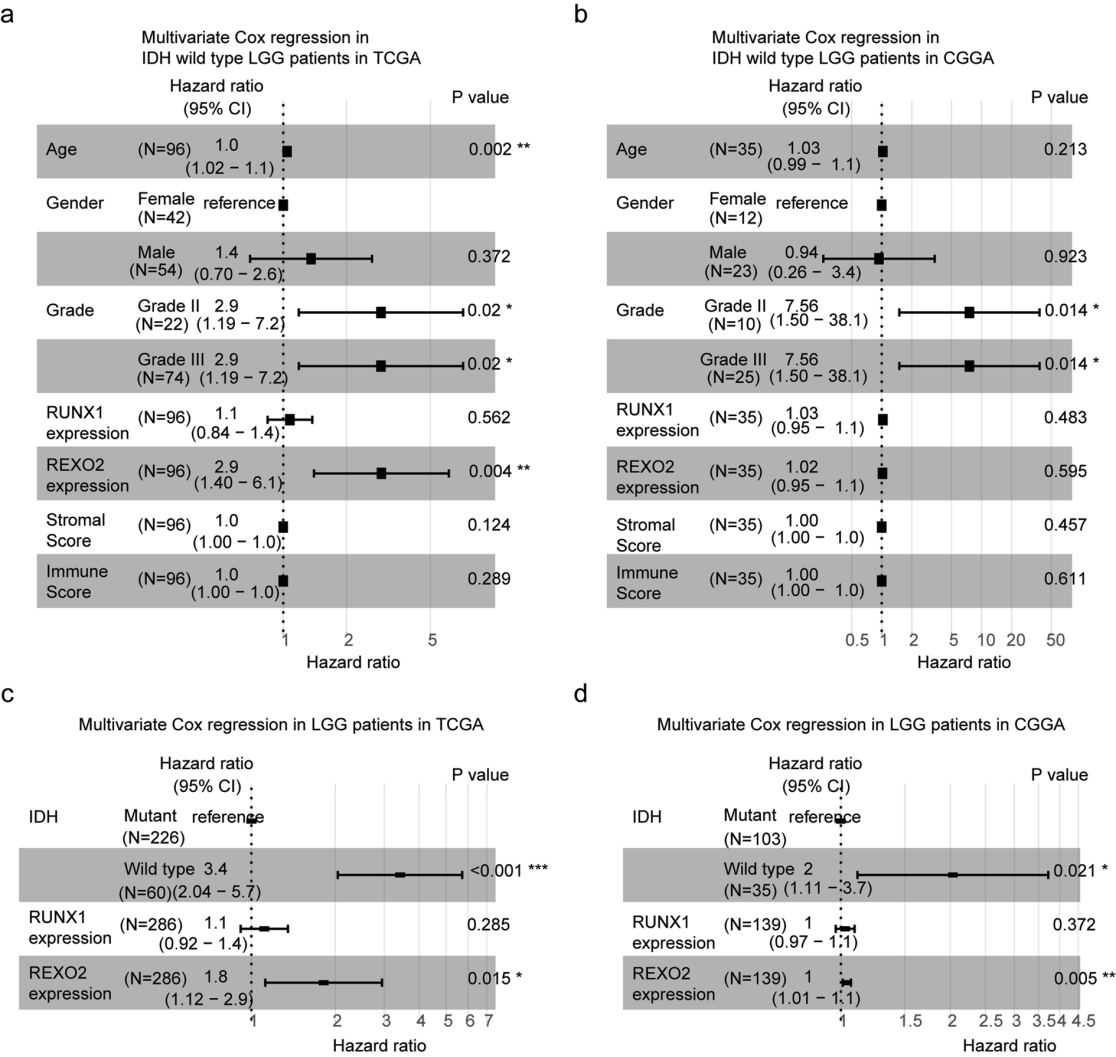
Age, tumor grade and REXO2 expression are independent prognostic factors in IDH wild type LGG patients. (**a**) Forest plot showed the correlations of age, gender, tumor grade, RUNX1 expression, REXO2 expression, stromal score, immune score with the clinical overall survival of IDH wild type LGG patients in TCGA dataset, determined by multivariate cox regression. (**b**) Forest plot showed the correlations of age, gender, tumor grade, RUNX1 expression, REXO2 expression, stromal score, immune score with the clinical overall survival of IDH wild type LGG patients in CGGA dataset. (**c**) Multivariate cox regression analysis determined the prognostic effects of IDH alteration, RUNX1 expression and REXO2 expression in LGG patients in TCGA dataset. (**d**) Multivariate cox regression analysis the prognostic effects of IDH alteration, RUNX1 expression and REXO2 expression in LGG patients in CGGA dataset.

All our results suggested that IDH wild type LGG was a heterogeneous cohort. In TCGA dataset, RUNX1 and REXO2 expressions were associated with the clinical outcomes of IDH wild type LGG patients. We then used multivariate cox regression survival analysis to detect the associations of the prognostic factors. The forest plots showed that age (*P* = 0.002), grade (*P* = 0.02) and REXO2 expression (*P* = 0.004) were independent prognostic markers in IDH wild type LGG patients in TCGA dataset (Fig. 8a). In CGGA dataset, tumor grade (*P* = 0.014) was an independent prognostic marker in IDH wild type LGG patients, while, age and REXO2 expression were not independent prognostic markers (*P* = 0.213 and *P* = 0.595, respectively) (Fig. 8b).

Furthermore, in TCGA and CGGA dataset, IDH alteration was an independent prognostic marker in LGG patients (*P* < 0.001 and *P* = 0.021, respectively) (Fig. 8c,d). Interestingly, although REXO2 expression was associated with IDH alterations, REXO2 expression was a prognostic marker independent IDH alterations in TCGA and CGGA datasets (*P* = 0.015 and *P* = 0.005, respectively) (Fig. 8c,d). Those results suggested that REXO2 was a good prognostic maker to predict the clinical outcomes of LGG.

## Discussion

IDH wild type LGG is a small subgroup of LGG. Only 20% LGG patients are without IDH mutations^2^. Because of the lack of enough samples, the inner heterogeneity within IDH wild type LGG subgroup is unclear. Using 96 IDH wild type LGG patients in TCGA and 35 IDH wild type LGG patients in CGGA dataset, we showed that IDH wild type LGG patients could divide into two sub-clusters by NMF assay. IDH wild type LGG patients in sub-cluster2 had prolonged overall survival and low frequency of CDKN2A alterations and low immune infiltrations. The prognostic relevance of CDKN2A loss was previously demonstrated in IDH mutant LGG^35,36^. IDH wild type LGG patients with higher immune alterations were associated with the worse prognosis. Those results were consistent with the classification of IDH wild type LGG patients by ConsensusClusterPlus method^28^, suggested that IDH wild type LGG was indeed a heterogeneous cohort, and could be further divided into two sub-clusters.

We further found that RUNX1 was correlated with the differentially expressed genes in sub-cluster1 IDH wild type LGG patients. Mutations of RUNX1 were occurred in nearly 10% acute myeloid leukemia (AML) patients^37–39^. Mutation of RUNX1 or high expression of RUNX1 in AML conferred the poor prognosis^40,41^. The expression of RUNX1 was also correlated with the clinical outcomes of lung adenocarcinomas^42^ and triple negative breast cancer^43^. Here, we showed that, RUNX1 was associated with the high immune infiltrations of IDH wild type LGG, and RUNX1 was associated with the worse clinical outcomes of IDH wild type or IDH mutant LGG patients. Moreover, RUNX1 expression and methylation were correlated with IDH mutation and the clinical outcomes of LGG or glioma patients. All those results highlighted the prognostic significance of RUNX1 in LGG. However, mutation of RUNX1 was barely detected in LGG^2^. The functions of RUNX1 in IDH wild type LGG should be studied.

REXO2 is a RNA binding protein and is required for maintaining the integrity of mitochondria^44,45^. Previous reports showed that higher expression levels of REXO2 were associated with the poor prognosis of prostate cancer^46^. However, the expression and prognosis of REXO2 in other malignant disease, particular in glioma were unknown. Here we showed that REXO2 was up-regulated in IDH wild type LGG patients with higher stromal score or immune score. And REXO2 expression and methylation were associated with the clinical outcomes of IDH wild type LGG patients. Moreover, REXO2 expression and methylation were associated with IDH mutation and the clinical outcomes of LGG or glioma patients. Furthermore, REXO2 expression was a prognostic marker independent IDH alteration, age and tumor grade, suggested that REXO2 was a good prognostic maker to predict the clinical outcomes of LGG.

Overall, our analysis highlighted the heterogeneity within IDH wild type LGG subgroup and revealed new prognostic factor of RUNX1 and REXO2 in IDH wild type LGG. However, the results were derived from published datasets and lack of further validations. Therefore, the expression and prognosis of RUNX1 and REXO2 in large cohorts of IDH wild type LGG should be studied. Also further studies of the functions of immune alterations in IDH wild type LGG are needed.

## Materials and methods

### Data collection

The gene expressions, DNA methylation along with the clinical datasets of TCGA were downloaded from https://tcga.xenahubs.net website. The CGGA datasets were downloaded from http://www.cgga.org.cn/index.jsp website.

### The nonnegative matrix factorization (NMF) classification

IDH wild type LGG patients in TCGA and CGGA datasets were classified into two sub-clusters using “NMF” package (version 0.22.0; https://cran.r-project.org/web/packages/NMF/index.html) based on the expression of all genes in R software^47^. Kaplan–Meier estimator determined the different clinical outcomes of IDH wild type LGG patients in sub-cluster1 and sub-cluster2. Log-rank test determined the *P* values.

### Heatmap presentation

Based on the criterion of absolute fold change > 2 and *P* values < 0.001, the differentially expressed genes between sub-cluster1 and sub-cluster2 of IDH wild type LGG patients were identified and were clustered using “pheatmap” package (version 1.0.12, https://cran.r-project.org/web/packages/pheatmap/) in R software.

### Stromal score, immune score and tumor purity

The stromal score, immune score and tumor purity of IDH wild type LGG were determined by “ESTIMATE” package based on the expression of all genes in R software. “ESTIMATE” package was downloaded from http://r-forge.r-project.org. ESTIMATE determined the stromal score, immune score and tumor purity based on single sample Gene Set Enrichment Analysis (ssGSEA)^48^. The stromal score and immune score were normalized using “scale” method in R. The normalized stromal score or immune score > 0 were defined as higher stromal score or immune score.

### Gene set enrichment analysis (GSEA) and ssGSEA

The enriched Kyoto Encyclopedia of Genes and Genomes (KEGG) signaling pathways and transcription factors in different sub-clusters of IDH wild type LGG patients were determined using GSEA (version 2.0)^49^. The GSEA software and gene sets were downloaded from www.broad.mit.edu/gsea/index.html. Totally, 186 KEGG pathways and 615 transcription factor gene sets were tested. Statistical *P* value was determined by 1,000 gene set permutations for GSEA analysis. *P* value < 0.05 was chosen to be significantly different. The regulatory impact of RUNX1 was determined using ssGSEA in “GSVA” package^50^ (version 4.0, http://www.bioconductor.org/packages/release/bioc/html/GSVA.html) in R.

### Survival analysis

The prognostic significance of RUNX1 and REXO2 were determined using Kaplan–Meier estimator in “survival” package (version 3.1-8, https://cran.r-project.org/web/packages/survival/index.html) in R statistics software. The prognostic effects of RUNX1 target genes were determined using univariate cox regression analysis in “survival” package. Log-rank test *P* value < 0.05 was chosen to be significantly different. The “high” and “low” subgroups were always defined based on the mean expression values of RUNX1 and REXO2. The hypo-methylation or hyper-methylation subgroups were always defined based on the mean methylation values of RUNX1 and REXO2.

### Forest plot

The forest plots were generated using “survival” and “survminer” packages “ggforest” method in R statistics software. The Hazard ratio and *P* values were determined using multivariate cox regression survival analysis. “survminer” package (version 0.4.2) was download from https://www.rdocumentation.org/.

### Statistical analysis

Box plots of the expression or methylation levels of RUNX1 and REXO2 were generated using GraphPad Prism 5.0. (https://www.graphpad.com/). *P* value was performed using the two tails paired Student’s t test. Contingency plots were also generated using GraphPad Prism 5.0. *P* value was performed using Chi-square test. *P* value < 0.05 was chosen to be significantly different.

## References

[1] Louis DN (2016). The 2016 World Health Organization classification of tumors of the central nervous system: A summary. Acta Neuropathol..

[2] Yan H (2009). IDH1 and IDH2 mutations in gliomas. N. Engl. J. Med..

[3] Parsons DW (2008). An integrated genomic analysis of human glioblastoma multiforme. Science.

[4] Brennan CW (2013). The somatic genomic landscape of glioblastoma. Cell.

[5] Dang L (2010). Cancer-associated IDH1 mutations produce 2-hydroxyglutarate. Nature.

[6] Duncan CG (2012). A heterozygous IDH1R132H/WT mutation induces genome-wide alterations in DNA methylation. Genome Res..

[7] Lu C (2012). IDH mutation impairs histone demethylation and results in a block to cell differentiation. Nature.

[8] Turcan S (2018). Mutant-IDH1-dependent chromatin state reprogramming, reversibility, and persistence. Nat. Genet..

[9] Philip B (2018). Mutant IDH1 promotes glioma formation in vivo. Cell Rep..

[10] Calvert AE (2017). Cancer-associated IDH1 promotes growth and resistance to targeted therapies in the absence of mutation. Cell Rep..

[11] Hartmann C (2013). Long-term survival in primary glioblastoma with versus without isocitrate dehydrogenase mutations. Clin. Cancer Res..

[12] Cancer Genome Atlas Research Network (2015). Comprehensive, integrative genomic analysis of diffuse lower-grade gliomas. N. Engl. J. Med..

[13] Nunez FJ (2019). IDH1-R132H acts as a tumor suppressor in glioma via epigenetic up-regulation of the DNA damage response. Sci. Transl. Med..

[14] Houillier C (2010). IDH1 or IDH2 mutations predict longer survival and response to temozolomide in low-grade gliomas. Neurology.

[15] Yin N (2020). IDH1-R132H mutation radiosensitizes U87MG glioma cells via epigenetic downregulation of TIGAR. Oncol. Lett..

[16] Wang XW (2014). IDH1(R132H) mutation increases U87 glioma cell sensitivity to radiation therapy in hypoxia. Biomed. Res. Int..

[17] Li S (2013). Overexpression of isocitrate dehydrogenase mutant proteins renders glioma cells more sensitive to radiation. Neuro Oncol..

[18] Koschmann C (2016). ATRX loss promotes tumor growth and impairs nonhomologous end joining DNA repair in glioma. Sci. Transl. Med..

[19] Ceccarelli M (2016). Molecular profiling reveals biologically discrete subsets and pathways of progression in diffuse glioma. Cell.

[20] Chan AK (2015). TERT promoter mutations contribute to subset prognostication of lower-grade gliomas. Mod. Pathol..

[21] Chan AK (2015). Combination genetic signature stratifies lower-grade gliomas better than histological grade. Oncotarget.

[22] Aibaidula A (2017). Adult IDH wild-type lower-grade gliomas should be further stratified. Neuro Oncol..

[23] Ma S (2020). Prognostic impact of CDKN2A/B deletion, TERT mutation, and EGFR amplification on histological and molecular IDH-wildtype glioblastoma. Neurooncol. Adv..

[24] Wilkerson MD, Hayes DN (2010). ConsensusClusterPlus: A class discovery tool with confidence assessments and item tracking. Bioinformatics.

[25] Wu F (2019). Molecular classification of IDH-mutant glioblastomas based on gene expression profiles. Carcinogenesis.

[26] Sadanandam A (2013). A colorectal cancer classification system that associates cellular phenotype and responses to therapy. Nat. Med..

[27] Wang H, Wang X, Xu L, Zhang J, Cao H (2019). A molecular sub-cluster of colon cancer cells with low VDR expression is sensitive to chemotherapy, BRAF inhibitors and PI3K-mTOR inhibitors treatment. Aging (Albany NY).

[28] Wu F (2020). Molecular subtyping reveals immune alterations in IDH wild-type lower-grade diffuse glioma. J. Pathol..

[29] Bao ZS (2014). RNA-seq of 272 gliomas revealed a novel, recurrent PTPRZ1-MET fusion transcript in secondary glioblastomas. Genome Res..

[30] Hu H (2018). Mutational landscape of secondary glioblastoma guides MET-targeted trial in brain tumor. Cell.

[31] Carro MS (2010). The transcriptional network for mesenchymal transformation of brain tumours. Nature.

[32] Ito Y, Bae SC, Chuang LS (2015). The RUNX family: Developmental regulators in cancer. Nat. Rev. Cancer.

[33] Otalora-Otalora BA, Henriquez B, Lopez-Kleine L, Rojas A (2019). RUNX family: Oncogenes or tumor suppressors (review). Oncol. Rep..

[34] Turcan S (2012). IDH1 mutation is sufficient to establish the glioma hypermethylator phenotype. Nature.

[35] Reis GF (2015). CDKN2A loss is associated with shortened overall survival in lower-grade (World Health Organization Grades II-III) astrocytomas. J. Neuropathol. Exp. Neurol..

[36] Appay R (2019). CDKN2A homozygous deletion is a strong adverse prognosis factor in diffuse malignant IDH-mutant gliomas. Neuro Oncol..

[37] Cancer Genome Atlas Research Network (2013). Genomic and epigenomic landscapes of adult de novo acute myeloid leukemia. N. Engl. J. Med..

[38] Mardis ER (2009). Recurring mutations found by sequencing an acute myeloid leukemia genome. N. Engl. J. Med..

[39] Papaemmanuil E (2016). Genomic classification and prognosis in acute myeloid leukemia. N. Engl. J. Med..

[40] Jalili M (2018). Prognostic value of RUNX1 mutations in AML: A Meta-Analysis. Asian Pac. J. Cancer Prev..

[41] Fu L (2016). High expression of RUNX1 is associated with poorer outcomes in cytogenetically normal acute myeloid leukemia. Oncotarget.

[42] Ramsey J (2018). Loss of RUNX1 is associated with aggressive lung adenocarcinomas. J. Cell Physiol..

[43] Ferrari N (2014). Expression of RUNX1 correlates with poor patient prognosis in triple negative breast cancer. PLoS ONE.

[44] Bruni F, Gramegna P, Oliveira JM, Lightowlers RN, Chrzanowska-Lightowlers ZM (2013). REXO2 is an oligoribonuclease active in human mitochondria. PLoS ONE.

[45] Nicholls TJ (2019). Dinucleotide degradation by REXO2 maintains promoter specificity in mammalian mitochondria. Mol. Cell.

[46] Hua X (2020). Effects of RNA binding proteins on the prognosis and malignant progression in prostate cancer. Front Genet.

[47] Gaujoux R, Seoighe C (2010). A flexible R package for nonnegative matrix factorization. BMC Bioinformatics.

[48] Hanzelmann S, Castelo R, Guinney J (2013). GSVA: Gene set variation analysis for microarray and RNA-seq data. BMC Bioinformatics.

[49] Subramanian A (2005). Gene set enrichment analysis: A knowledge-based approach for interpreting genome-wide expression profiles. Proc. Natl. Acad. Sci. USA.

[50] Yoshihara K (2013). Inferring tumour purity and stromal and immune cell admixture from expression data. Nat. Commun..

